# Large-scale bidirectional Mendelian randomization study identifies new gut microbiome significantly associated with immune thrombocytopenic purpura

**DOI:** 10.3389/fmicb.2024.1423951

**Published:** 2024-07-04

**Authors:** Jiawei Li, Jia Li, Yuxiao Liu, Juanhuan Zeng, Yuan Liu, Yeke Wu

**Affiliations:** ^1^School of Basic Medical Sciences, Chengdu University of Traditional Chinese Medicine, Chengdu, China; ^2^Department of Stomatology, Hospital of Chengdu University of Traditional Chinese Medicine, Chengdu, China

**Keywords:** Henoch-Schönlein purpura, immune thrombocytopenic purpura, Mendelian randomization, autoimmune diseases, gut microbiota

## Abstract

**Introduction:**

A variety of studies have shown a link between the gut microbiota and autoimmune diseases, but the causal relationship with Henoch-Schönlein purpura (HSP) and immune thrombocytopenic purpura (ITP) is unknown.

**Methods:**

This study investigated the bidirectional causality between gut microbiota and HSP and ITP using Mendelian randomization (MR). Large-scale genetic data of gut microbiota at phylum to species level from the MiBioGen consortium and the Dutch Microbiome Project were utilized. Genome-wide association studies (GWAS) summary statistics for HSP and ITP came from FinnGen R10. Various MR methods were applied to infer causal relationships, including inverse variance weighted (IVW), maximum likelihood (ML), cML-MA, MR-Egger, weighted median, weighted model, and MR-PRESSO. Multiple sensitivity analyses and Bonferroni correction were conducted to enhance robustness and reliability.

**Results:**

Based on the IVW estimates, 23 bacterial taxa were identified to have suggestive associations with HSP and ITP. Remarkably, after Bonferroni correction, family *Alcaligenaceae* (OR = 2.86, 95% CI = 1.52–5.37; IVW, *p* = 1.10 × 10^−3^, ML, *p* = 1.40 × 10^−3^) was significantly associated with ITP as a risk factor, while family *Bacteroidales S24 7group* (OR = 0.46, 95% CI = 0.29–0.74; IVW, *p* = 1.40 × 10^−3^) was significantly associated with ITP as a protective factor. No significant associations between HSP and ITP and gut microbiota were found in reverse analyses.

**Conclusion:**

Our study provides evidence of causal effects of gut microbiota on HSP and ITP, highlighting the importance of further research to clarify the underlying mechanisms and develop targeted therapeutic interventions for these autoimmune diseases.

## Introduction

HSP and ITP are characterized by purpura, resulting from small blood vessel bleeding into the skin and mucous membranes (Rodeghiero et al., 2009; Hetland et al., 2017). HSP, also known as IgA vasculitis, presents as a systemic vasculitis with palpable purpura, arthritis, gastrointestinal symptoms, and kidney involvement, while ITP manifests as thrombocytopenia due to immune-mediated platelet destruction, predisposing individuals to bleeding (Piel-Julian et al., 2018; Hahn et al., 2023). Despite their distinct clinical presentations, both disorders share common immunopathogenic mechanisms, hinting at potential etiological similarities (Zufferey et al., 2017). Children affected by HSP and ITP endure significant morbidity, including physical suffering and treatment-related adverse effects such as corticosteroid-induced weight gain and mood changes, which can cause psychological distress and impair overall health and quality of life (Kühne et al., 2003; Terrell et al., 2010; Weiss et al., 2010).

The human gut microbiota, which comprises a vast number of microorganisms residing in the gastrointestinal tract, is essential for supporting the immune system and metabolic balance in the host (Postler and Ghosh, 2017). Dysbiosis, or alterations in gut microbiota composition and function, has been implicated in various immune-mediated disorders, including HSP and ITP (Arrieta et al., 2014). Observational studies indicate a potential association between gut microbiota and HSP and ITP. For instance, Wang et al. (2018) found a significant negative association between IgA levels and *Bifidobacterium* in HSP children. Zhang et al. (2021) found that *Escherichia Shigella* has diagnostic value for HSP recurrence. Li et al. (2022) found that *Proteobacteria* and *Actinobacteria* are associated with organ involvement in HSP. In addition, Borody et al. (2011) reported a case of an ITP patient whose platelet levels returned to normal after receiving fecal microbiota transplantation. Further research on the gut microbiome could potentially aid in the management and therapy of HSP and ITP. However, establishing causality in such studies is challenging due to confounding factors and the potential for reverse causation (Rinninella et al., 2019).

MR offers a robust methodological approach to infer causal relationships and reduce confounding and reverse causation biases by utilizing genetic variants as instrumental variables (IVs), which are randomly allocated at conception and remain unchanged throughout life (Davies et al., 2018). To achieve valid results, MR analysis needs to satisfy three core IVs assumptions: (I) Relevance assumption: Genetic variations are closely associated with the exposure factor; (II) Independence assumption: Genetic variations are independent of any known or unknown confounding factors; (III) Exclusion restriction assumption: Genetic variations only affect the outcome through the exposure factor and not through any other direct causal pathways (Davey Smith and Hemani, 2014). In this study, we aim to investigate the bidirectional causal relationship between gut microbiota and HSP and ITP using a two-sample MR method. By integrating large-scale genetic data and summary statistics from GWAS of gut microbiota composition and HSP and ITP susceptibility, we will assess whether genetic predisposition to gut microbiota dysbiosis influences the risk of HSP and ITP, and conversely, whether genetic susceptibility to HSP and ITP impacts gut microbiota composition.

## Methods

### Study design and data sources

**Figure 1:**
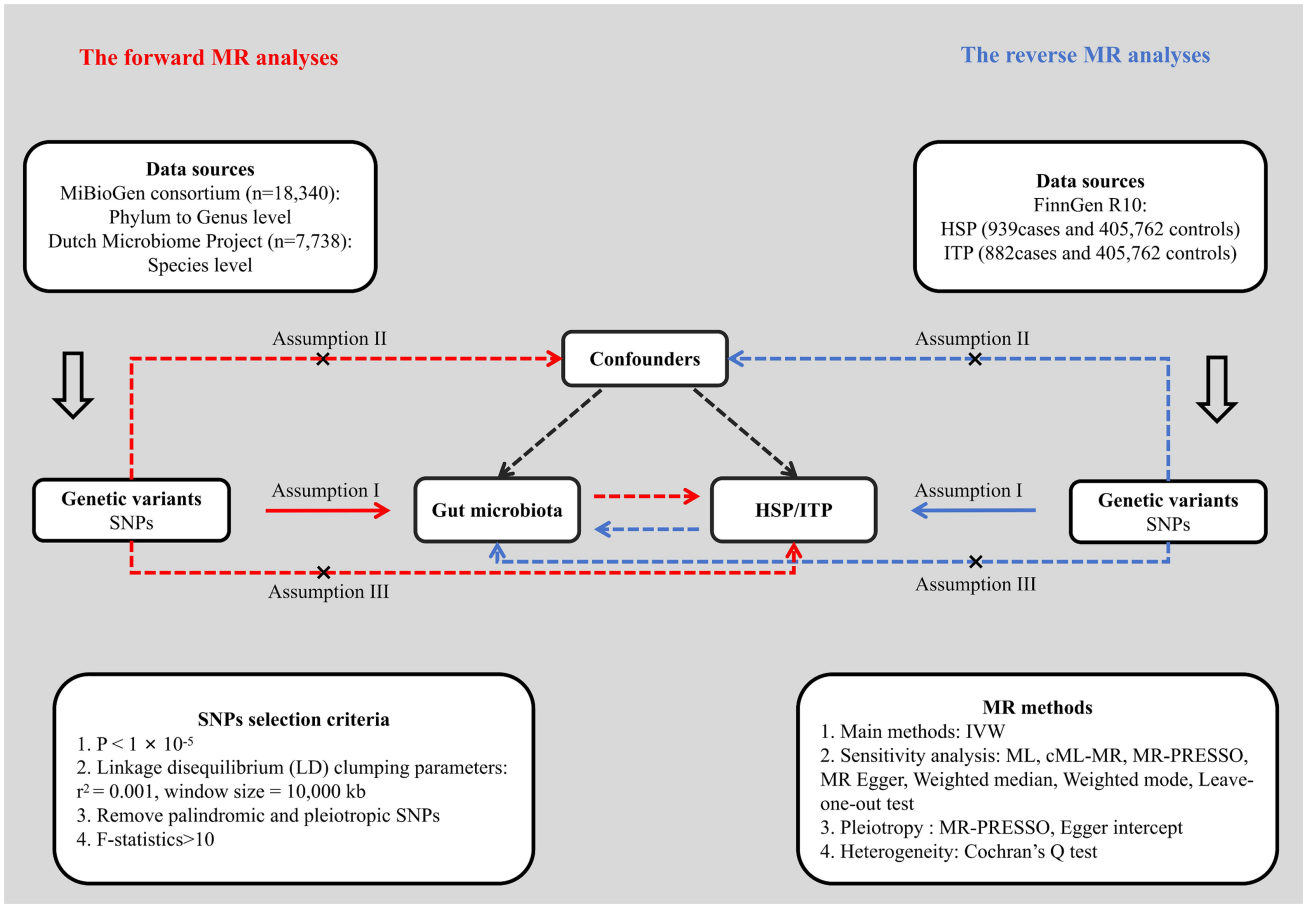
Flowchart of the study design. HSP, Henoch-Schönlein purpura; ITP, immune thrombocytopenic purpura; SNP, single nucleotide polymorphism; IVW, inverse variance weighted; ML, maximum likelihood; MR, Mendelian randomization.

We first performed a bidirectional two-sample MR analysis to examine the causality between gut microbiota and HSP and ITP, employing single nucleotide polymorphisms (SNPs) obtained from GWAS summary statistics as IVs. Subsequently, various sensitivity analysis methods and multiple corrections were applied to enhance the robustness and reliability of the study results. This study utilized publicly available GWAS summary statistics, with ethical approvals obtained from the original studies. The flowchart of the study design is shown in Figure 1.

#### Gut microbiota

In this MR study, species represents the lowest taxonomic level of gut microbiota. The MiBioGen consortium conducted the most comprehensive genome-wide meta-analysis on gut microbiota composition and provides the genetic variations for gut microbiota at the phylum to genus levels (Kurilshikov et al., 2021). This study includes data from 24 cohorts and 18,340 individuals, with 16 cohorts and 13,266 individuals being of single European ancestry. Microbial composition profiling was done by clustering different regions of the 16S rRNA gene: V4, V3-V4, and V1-V2, with taxonomic classification completed by direct taxonomic binning. This study involved analyzing 211 different taxa in the gut microbiota and carried out a microbiota quantitative trait loci (mbQTL) mapping analysis to determine how host genetics impact the abundance of these bacterial species. After removing 15 unknown identified bacterial taxa, the MR analysis included taxa from 9 phyla, 16 classes, 20 orders, 32 families, and 119 genera. GWAS summary statistics for gut microbiota at species level were derived from the Dutch Microbiome Project (Lopera-Maya et al., 2022). This dataset comprised genetic variants of 105 species with 7,738 European individuals.

#### HSP and ITP

GWAS summary statistics for HSP (939 cases and 405,762 controls) and ITP (882 cases and 405,762 controls) were obtained from FinnGen R10 dataset, which was released in December 2023, with 412,181 samples (230,310 females and 181,871 males) and 21,311,942 genetic variants. In this study, a total of 2,408 phenotypes were constructed for GWAS analysis using the International Classification of Diseases, 10th revision (ICD-10) diagnostic code. FinnGen research project is a large-scale genomics initiative combining genotype data from over 500,000 Finnish biobank samples and digital health record data from Finnish health registries, aiming to provide new insights into the genetics of disease (Kurki et al., 2023).

### Selection of instrumental variables

For gut microbiota, a loose significance threshold (*p* < 1× 10^−5^) was applied to guaranteed sufficient SNPs for further MR Analysis (Sanna et al., 2019). The linkage disequilibrium (LD) clumping was conducted by utilizing European samples data from the 1,000 Genomes project as the reference panel, and the parameters were set as r^2^ = 0.001 and window size = 10,000 kb. Only SNPs with minor allele frequency (MAF) > 0.01 and lowest *p*-value were retained. Palindromic and potentially pleotropic SNPs were excluded by conducting a harmonization procedure and searching the PhenoScanner database (Staley et al., 2016). To prevent bias from weak instruments, we calculated the F-statistic to assess the strength of each SNP. The formula used was $F=\frac{R^{2}x\left(N-2\right)}{1-R^{2}}$ , with R^2^ denoting the percentage of variability in exposure factors explained by the genetic variations and N denoting the sample size (Burgess and Thompson, 2011). SNPs with F-statistics below 10 have been excluded.

### Statistical analysis

In our study, a variety of MR approaches were utilized to investigate the causality between gut microbiota and HSP and ITP, including IVW, ML, cML-MA, MR-Egger, weighted median, weighted model and MR-PRESSO. IVW was primarily used to establish causality, while the other methods served as sensitivity analyses to ensure robustness. In a meta-analysis model, IVW combines the ratio estimates from each variant to provide an overall estimate of the causal effect (Burgess et al., 2015b). Based on the hypothesis of IVW, there should be no pleiotropy in IVs, otherwise the results would be biased (Burgess et al., 2015a). ML is similar to IVW and helps to estimate bias due to sample overlap (Burgess et al., 2013). MR-Egger differs from IVW in its evaluation of pleiotropy, by taking into account the presence of an intercept term. If this intercept term approaches zero, the MR-Egger regression model closely resembles IVW. However, significant deviation indicates potential horizontal pleiotropy among IVs (Bowden et al., 2015, 2016b). cML-MA, a powerful MR approach, was used to mitigate bias from correlated and uncorrelated pleiotropy and was considered much more accurate than MR-Egger (Yin and Zhu, 2023). Weighted median provides reliable estimates even when up to 50% of the IVs are invalid, while weighted mode reduces bias and type-I errors in many situations and exhibits greater power than MR-Egger (Bowden et al., 2016a; Hartwig et al., 2017). MR-PRESSO, including global test and outlier test, was utilized to detect horizontal pleiotropy and remove outliers using bacterial taxa with at least three valid SNPs (Verbanck et al., 2018). Cochran’s Q test and leave-one-out analysis were used for detection of potential heterogeneity in IVW method. Additionally, reverse MR analysis was conducted for bacterial taxa identified as causally related to HSP and ITP, with parameters and methods consistent with the forward analysis. The statistical power for all MR estimates was assessed using Stephen Burgess’ online calculator tool (Brion et al., 2012; Burgess, 2024). This tool helped in assessing the power for detecting significant associations, ensuring that the study was adequately powered to detect the expected effects (Table 1).

**Table 1:** MR estimates for the causal association between identified bacterial taxa and ITP.

Bacterial taxa (exposure)	MR method	No. SNP	*P*	OR	95% CI	F-statistic	Power
*Family Alcaligenaceae*	IVW	9	**0.0011**	2.86	1.52–5.37	19.78	1.00
	ML	9	**0.0014**	2.94	1.52–5.68		
	cML-MA	9	0.0036	3.00	1.43–6.3		
*Family Bacteroidales S24 7group*	IVW	7	**0.0014**	0.46	0.29–0.74	43.63	0.65
	ML	7	0.0018	0.46	0.28–0.75		
	cML-MA	7	0.0022	0.45	0.27–0.75		
*Genus Allisonella*	IVW	8	0.0239	0.68	0.49–0.95	98.49	0.59
	ML	8	0.0062	0.67	0.5–0.89		
	cML-MA	8	0.0161	0.67	0.48–0.93		
*Genus Coprococcus2*	IVW	8	0.0299	0.55	0.32–0.94	27.63	0.39
	ML	8	0.0309	0.54	0.31–0.95		
	cML-MA	8	0.0328	0.52	0.28–0.95		
*Genus Escherichia Shigella*	IVW	10	0.0491	1.68	1–2.82	28.07	0.82
	ML	10	0.0467	1.71	1.01–2.9		
	cML-MA	10	0.0706	1.70	0.96–3.03		
*Genus Eubacterium hallii group*	IVW	14	0.0381	0.64	0.41–0.98	25.15	0.40
	ML	14	0.0466	0.64	0.41–0.99		
	cML-MA	14	0.1033	0.63	0.36–1.1		
*Genus Eubacterium ruminantium group*	IVW	17	0.0422	0.74	0.56–0.99	43.83	0.43
	ML	17	0.0498	0.75	0.56–1		
	cML-MA	17	0.0560	0.75	0.56–1.01		
*Species Odoribacter splanchnicus*	IVW	14	0.0081	1.45	1.1–1.9	30.44	0.87
	ML	14	0.0085	1.46	1.1–1.93		
	cML-MA	14	0.0112	1.50	1.1–2.05		
*Species Dialister invisus*	IVW	6	0.0176	0.67	0.49–0.93	58.79	0.57
	ML	6	0.0184	0.66	0.47–0.93		
	cML-MA	6	0.0326	0.68	0.47–0.97		
*Species Bilophila unclassified*	IVW	11	0.0198	0.66	0.46–0.94	25.11	0.49
	ML	11	0.0240	0.66	0.46–0.95		
	cML-MA	11	0.0496	0.67	0.44–1		
*Species Haemophilus parainfluenzae*	IVW	6	0.0180	0.65	0.46–0.93	71.96	0.71
	ML	6	0.0051	0.64	0.46–0.87		
	cML-MA	6	0.0186	0.65	0.45–0.93		
*Species Bacteroides finegoldii*	IVW	16	0.0076	0.81	0.7–0.95	98.82	0.74
	ML	16	0.0091	0.81	0.69–0.95		
	cML-MA	16	0.0349	0.81	0.67–0.99		
*Species Dorea unclassified*	IVW	10	0.0198	0.76	0.61–0.96	82.33	0.64
	ML	10	0.0075	0.75	0.61–0.93		
	cML-MA	10	0.0335	0.75	0.58–0.98		

SNP, single nucleotide polymorphism; IVW, inverse variance weighted; ML, maximum likelihood; OR, odds ratio; CI, confidence interval. P-values that satisfy the Bonferroni correction are bolded.

In order to enhance the thorough evaluation of causality, a Bonferroni correction was utilized to set separate significance thresholds across various taxonomic tiers (Curtin and Schulz, 1998). The thresholds were established considering the quantity of bacteria at every taxonomic level and are presented as follows: 5.6 × 10^−3^ (0.05/9, phylum), 3.1 × 10^−3^ (0.05/16, class), 2.5 × 10^−3^ (0.05/20, order), 1.6 × 10^−3^ (0.05/32, family), 4.2 × 10^−4^ (0.05/119, genus), and 5.0 × 10^−4^ (0.05/105, species). *p*-value less than 0.05 but greater than the corrected threshold was considered an indication of a suggestive causal association.

All MR analyses were conducted in R software (version 4.3.0) using the “MR-PRESSO” (version 1.0), “TwoSampleMR” (version 0.5.7) and “MRcML” (version 0.0.0.9) R packages.

## Results

### Causal links between gut microbiota and HSP

**Figure 2:**
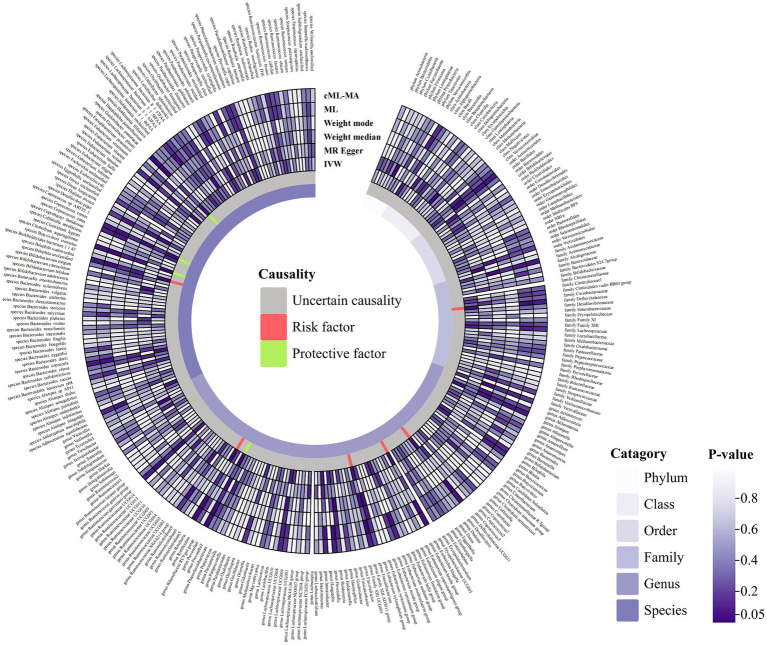
Circular hotspot map of causal effects of gut microbiota on HSP represented by all MR estimates. IVW, inverse variance weighted; ML, maximum likelihood. Created with https://www.chiplot.online/.

Following the selection criteria, a total of 3,010 SNPs in 9 taxa at phylum level, 16 taxa at class level, 20 taxa at order level, 31 taxa at family level, 117 taxa at genus level and 101 taxa at species level were used as IVs for the MR analysis of gut microbiota on HSP. All MR estimates and details about IVs of the causal effects of gut microbiota on HSP are shown in Supplementary Tables S1, S2. Based on the IVW estimates, family *Defluviitaleaceae* (*p* = 4.32 × 10^−2^, OR = 1.52), genus *Defluviitaleaceae UCG01* (*p* = 3.53 × 10^−2^, OR = 1.65), genus *Escherichia Shigella* (*p* = 4.03 × 10^−2^, OR = 1.69), genus *Family XIII AD3011 group* (*p* = 3.81 × 10^−2^, OR = 1.93), genus *Roseburia* (*p* = 2.42 × 10^−2^, OR = 1.78) and species *Bilophila wadsworthia* (*p* = 2.87 × 10^−2^, OR = 1.55) were associated with HSP as risk factors, while genus *Prevotella9* (*p* = 2.09 × 10^−2^, OR = 0.66), species *Gordonibacter pamelaeae* (*p* = 4.61 × 10^−2^, OR = 0.77), species *Clostridium asparagiforme* (*p* = 2.94 × 10^−2^, OR = 0.85) and species *Coprococcus comes* (*p* = 4.30 × 10^−2^, OR = 0.72) were associated with HSP as protective factors (Table 2; Figure 2; Supplementary Figure S1) However, after Bonferroni correction, the above MR estimates only could reveal suggestive associations between these 10 bacterial taxa and HSP. According to the results of the MR-PRESSO (Supplementary Table S9) and MR-Egger intercept term (Supplementary Table S7), there were no significant horizontal pleiotropy and outliers. In addition, Cochran’s Q test (Supplementary Table S5) and leave-one-out analysis (Supplementary Figure S3) found no potentially heterogeneous IVs. The reverse analysis revealed no significant causality between HSP and gut microbiota (Supplementary Table S11).

**Table 2:** MR estimates for the causal association between identified bacterial taxa and HSP.

Bacterial taxa (exposure)	MR method	No. SNP	*P*	OR	95% CI	F-statistic	Power
*Family Defluviitaleaceae*	IVW	11	0.0432	1.52	1.01–2.27	39.18	0.78
	ML	11	0.0353	1.54	1.03–2.31		
	cML-MA	11	0.0786	1.47	0.96–2.27		
*Genus Defluviitaleaceae UCG011*	IVW	9	0.0353	1.65	1.04–2.63	41.28	0.93
	ML	9	0.0238	1.69	1.07–2.66		
	cML-MA	9	0.0538	1.65	0.99–2.74		
*Genus Escherichia Shigella*	IVW	10	0.0403	1.69	1.02–2.79	28.07	0.85
	ML	10	0.0405	1.71	1.02–2.84		
	cML-MA	10	0.0555	1.72	0.99–2.98		
*Genus Family XIII AD3011 group*	IVW	13	0.0381	1.93	1.04–3.6	23.33	0.98
	ML	13	0.0042	2.01	1.25–3.24		
	cML-MA	13	0.0870	1.80	0.92–3.51		
*Genus Prevotella9*	IVW	15	0.0209	0.66	0.47–0.94	36.36	0.55
	ML	15	0.0172	0.66	0.47–0.93		
	cML-MA	15	0.0299	0.62	0.41–0.96		
*Genus Roseburia*	IVW	13	0.0242	1.78	1.08–2.93	20.82	0.92
	ML	13	0.0236	1.80	1.08–3.01		
	cML-MA	13	0.0300	1.80	1.06–3.06		
*Species Gordonibacter pamelaeae*	IVW	4	0.0461	0.77	0.6–1	158.25	0.51
	ML	4	0.0169	0.76	0.61–0.95		
	cML-MA	4	0.0265	0.76	0.59–0.97		
*Species Clostridium asparagiforme*	IVW	13	0.0294	0.85	0.74–0.98	127.23	0.56
	ML	13	0.0277	0.85	0.73–0.98		
	cML-MA	13	0.0618	0.85	0.72–1.01		
*Species Bilophila wadsworthia*	IVW	7	0.0287	1.55	1.05–2.29	30.17	0.83
	ML	7	0.0296	1.57	1.05–2.35		
	cML-MA	7	0.0529	1.52	0.99–2.31		
*Species Coprococcus comes*	IVW	9	0.0430	0.72	0.53–0.99	34.45	0.40
	ML	9	0.0442	0.72	0.52–0.99		
	cML-MA	9	0.0822	0.72	0.5–1.04		

SNP, single nucleotide polymorphism; IVW, inverse variance weighted; ML, maximum likelihood; OR, odds ratio; CI, confidence interval.

### Causal links between gut microbiota and ITP

**Figure 3:**
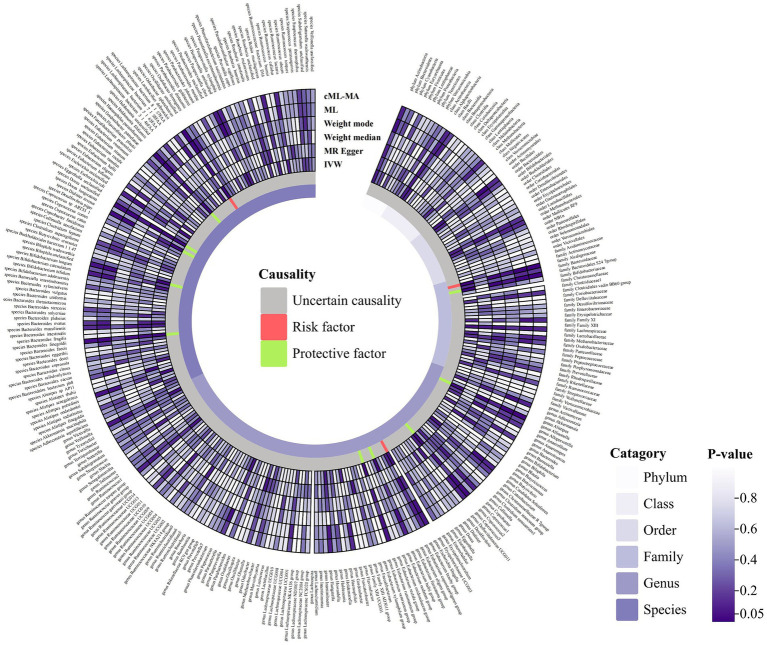
Circular hotspot map of causal effects of gut microbiota on ITP represented by all MR estimates. IVW, inverse variance weighted; ML, maximum likelihood. Created with https://www.chiplot.online/.

A total of 2,993 SNPs in 9 taxa at phylum level, 16 taxa at class level, 20 taxa at order level, 31 taxa at family level, 117 taxa at genus level and 101 taxa at species level were used as IVs for the MR analysis of gut microbiota on ITP with the same selection criteria. All MR estimates and details about IVs of the causal effects of gut microbiota on ITP are shown in Supplementary Tables S3, S4. Based on the IVW estimates, family *Alcaligenaceae* (*p* = 1.10 × 10^−3^, OR = 2.86), genus *Escherichia Shigella* (*p* = 4.91 × 10^−2^, OR = 1.68) and species *Odoribacter splanchnicus* (*p* = 8.10 × 10^−3^, OR = 1.45) were associated with ITP as risk factors, while family *Bacteroidales S24 7group* (*p* = 1.46 × 10^−3^, OR = 0.46), genus *Allisonella* (*p* = 2.39 × 10^−2^, OR = 0.68), genus *Coprococcus2* (*p* = 2.99 × 10^−2^, OR = 0.55), genus *Eubacterium hallii group* (*p* = 3.81 × 10^−2^, OR = 0.64), genus *Eubacterium ruminantium group* (*p* = 4.22 × 10^−2^, OR = 0.74), species *Dialister invisus* (*p* = 1.76 × 10^−2^, OR = 0.67), species *Bilophila unclassified* (*p* = 1.98 × 10^−2^, OR = 0.66), species *Haemophilus parainfluenzae* (*p* = 1.80 × 10^−2^, OR = 0.65), species *Bacteroides finegoldii* (*p* = 7.60 × 10^−3^, OR = 0.81) and species *Dorea unclassified* (p = 1.98 × 10^−2^, OR = 0.76) were associated with ITP as protective factors (Table 1; Figure 3; Supplementary Figure S2). Notably, after Bonferroni correction, family *Alcaligenaceae* (IVW, p = 1.10 × 10^−3^; ML, *p* = 1.40 × 10^−3^) and family *Bacteroidales S24 7group* (IVW, p = 1.40 × 10^−3^) still remained significantly associated with ITP. No significant horizontal pleiotropy and outliers were detected by MR-PRESSO (Supplementary Table S10) and MR-Egger intercept term (Supplementary Table S8). Furthermore, Cochran’s Q test (Supplementary Table S6) and leave-one-out analysis (Supplementary Figure S4) did not identify any potentially heterogeneous IVs. The reverse analysis revealed no significant causality between ITP and gut microbiota (Supplementary Table S11).

## Discussion

This study comprehensively evaluates the causal relationship between gut microbiota and two autoimmune diseases (HSP and ITP) from the perspective of genetic variation using a two-sample MR approach. Indeed, previous MR studies have extensively explored the relationship between the gut microbiota and autoimmune diseases such as type 1 diabetes, multiple sclerosis, Sjögren syndrome, systemic lupus erythematosus and inflammatory bowel disease (Liu et al., 2022; Xu et al., 2022; Cao et al., 2023; Luo et al., 2023). MR estimates revealed suggestive associations between specific bacterial taxa and HSP, specifically, *Defluviitaleaceae*, genus *Defluviitaleaceae UCG01*, genus *Escherichia Shigella*, genus *Family XIII AD3011 group*, genus *Roseburia* and species *Bilophila wadsworthia* are suggestively associated with increased risk of HSP, whereas genus *Prevotella9*, species *Gordonibacter pamelaeae*, species *Clostridium asparagiforme* and species *Coprococcus comes* are suggestively protective against HSP. Similarly, MR analysis demonstrated that family *Alcaligenaceae* and family *Bacteroidales S24 7group* had significant associations with ITP after strict correction, with the former acting as a risk factor and the latter as a protective factor. In addition, genus *Allisonella*, genus *Coprococcus2*, genus *Eubacterium hallii group*, genus *Eubacterium ruminantium group*, species *Dialister invisus*, species *Bilophila unclassified*, species *Haemophilus parainfluenzae*, species *Bacteroides finegoldii* and species *Dorea unclassified* had suggestive protective effects on ITP, while genus *Escherichia Shigella* and species *Odoribacter splanchnicus* had suggestive risk effects on ITP. It is worth noting that for the two significant associations mentioned above, the statistical power is 100 and 65%, respectively, indicating robust statistical significance at the 0.05 *p*-value threshold.

In addition to establishing the causal links between gut microbiota composition and HSP and ITP, it is imperative to explore the potential mechanisms whereby the gut microbiota affects these autoimmune disorders. The gut microbiota is involved in shaping host immune responses and maintaining immune homeostasis through various mechanisms, including modulation of the intestinal barrier function, production of metabolites, and interaction with the host immune system (Belkaid and Hand, 2014; Rooks and Garrett, 2016). Alterations in composition and function of gut microbiota can dysregulate these regulatory mechanisms, leading to aberrant immune activation and autoimmunity (Wu and Wu, 2012; Manfredo Vieira et al., 2018). One potential mechanism by which the gut microbiota may contribute to the pathogenesis of HSP and ITP is through the modulation of mucosal barrier integrity (Saps et al., 2010). Intestinal epithelial cells create a physical barrier that separates luminal contents, including commensal microbes, from the underlying immune cells in the gut-associated lymphoid tissue (Abraham and Medzhitov, 2011). Dysbiosis-induced impairment of the intestinal barrier can lead to increased permeability, allowing translocation of microbial products and activation of mucosal immune responses (Buford et al., 2018). This chronic mucosal inflammation may promote systemic immune dysregulation and contribute to the development of autoimmune diseases such as HSP and ITP. Moreover, the gut microbiota plays a vital role in producing various metabolites, such as short-chain fatty acids (SCFAs), bile acids, and secondary metabolites derived from dietary components. These microbial metabolites can function as signaling molecules that regulate host immune function and inflammatory responses. SCFAs, mainly produced by gut bacteria through the fermentation of dietary fiber, have been demonstrated to have anti-inflammatory properties by promoting regulatory T cell differentiation and inhibiting pro-inflammatory cytokine production (den Besten et al., 2013; Park et al., 2015; Tan et al., 2016). Dysbiosis-induced changes in SCFA production may disrupt immune tolerance and promote autoimmune pathology in HSP and ITP. Specific bacterial taxa in the gut microbiota may interact directly with the host immune system, either by eliciting immune responses or by influencing immune cell development and function (Zhang et al., 2017). Certain pathogenic bacteria, such as *Escherichia coli* and *Shigella* species, have been linked to the development of autoimmune diseases through molecular mimicry. This occurs when microbial antigens resemble host antigens, leading to cross-reactive immune responses. In contrast, commensal bacteria, such as those found in the *Clostridiales* order, have been demonstrated to stimulate the production of regulatory T cells and promote immune tolerance, thereby providing protection against autoimmunity (Tremlett and Waubant, 2018; Zitvogel et al., 2018). It should be noted that the gut microbiota can also affect systemic inflammation and coagulation pathways, which may contribute to the vascular and hematological manifestations observed in HSP and ITP (Wang et al., 2021). Dysbiosis-induced inflammation and endothelial dysfunction may enhance platelet activation and aggregation, leading to purpura and thrombocytopenia, which are characteristic of HSP and ITP.

The gut microbiota has a multifaceted impact on host immunity and inflammatory pathways, influencing the pathogenesis of autoimmune diseases such as HSP and ITP (Aminov et al., 2006). Findings from this study suggest that specific gut microbiota compositions are associated with HSP and ITP. For instance, the family *Alcaligenaceae* has been identified as a risk factor for ITP, while the family *Bacteroidales S24 7group* appears to be a protective factor. These insights can inform the development of microbiota-based diagnostics, enabling earlier detection and intervention in clinical settings.

Leveraging the causal relationships established in this study, clinicians could adopt microbiota-targeted therapies, such as probiotics, prebiotics, or fecal microbiota transplantation, as part of a comprehensive treatment plan for patients with HSP and ITP. This approach could help in modulating the immune response, reducing inflammation, and potentially restoring immune balance more rapidly than current treatments, which primarily focus on symptom management rather than addressing underlying causes.

A recent study by Jiang et al. (2024) preliminarily explored the causal relationship between gut microbiota and HSP and ITP using MR. However, this study has several significant differences and advantages. First, in terms of data sources, the gut microbiota involved in this study was more comprehensive, covering the species level, and the R10 database had a larger sample size and more SNPs. More importantly, there is a significant discrepancy between the results of the two studies, which may be precisely the consequence of different data sources. IVs are crucial in MR analyses, and therefore MR estimates obtained by analyzing more recent and larger databases tend to be more representative of potential causal associations. Second, the positive results were supported by at least three powerful MR methods (IVW, ML, cML-MA) and met the criteria for Bonferroni correction. These measures reduced the likelihood of false-positive results and increased robustness. This study also has limitations. The genetic makeup of European populations differs from that of other ancestries. This lack of diversity can result in findings that are not generalizable to other populations. There are known geographical differences in gut microbiota composition due to diet, lifestyle, and other environmental factors. Findings based on European populations might not apply to other regions with different microbiota profiles. Differences in ancestry can lead to population stratification, which can confound the results if not properly adjusted for in the analysis. Factors such as age, body mass index, diet, and lifestyle are critical confounders in studies of gut microbiota. While MR aims to reduce these biases, complete control over all confounding factors is challenging. It is crucial for future studies to include more diverse populations to enhance the generalizability of the results.

## Conclusion

Our study identified suggestive causal effects of gut microbiota on HSP and ITP. Particularly, family *Alcaligenaceae* and family *Bacteroidales S24 7group* have a significant association with ITP after Bonferroni correction. Further research using advanced molecular and immunological techniques is necessary to understand the complex interactions between the gut microbiota, host immune system, and autoimmune pathogenesis in HSP and ITP.

## Data availability statement

Publicly available datasets were analyzed in this study. GWAS summary statistics for mbQTLs are available at www.mibiogen.org. The original contributions presented in the study are included in the article/Supplementary material, further inquiries can be directed to the corresponding author/s.

## Author contributions

JiawL: Writing – review & editing, Writing – original draft, Methodology, Formal analysis, Data curation, Conceptualization. JiaL: Writing – review & editing, Writing – original draft, Software, Investigation, Formal analysis. YuxL: Writing – review & editing, Writing – original draft, Visualization, Validation, Supervision. JZ: Writing – review & editing, Writing – original draft, Visualization, Validation, Supervision. YuaL: Writing – review & editing, Writing – original draft, Resources, Project administration. YW: Writing – review & editing, Writing – original draft, Resources, Project administration, Funding acquisition.
