# Supplementary material for: Large-scale bidirectional Mendelian randomization study identifies new gut microbiome significantly associated with immune thrombocytopenic purpura
Source: Front Microbiol. 2024 Jul 4;15:1423951. doi: 10.3389/fmicb.2024.1423951 (PMC11257036; doi:10.3389/fmicb.2024.1423951)
Supplement: Supplementary file 1 [file Data_Sheet_1.pdf]

# Supplementary Figures

**Figure S1. Scatter plots for causal effect of gut microbiota on HSP.....................2**

**Figure S2. Scatter plots for causal effect of gut microbiota on ITP.......................3**

**Figure S3. MR leave-one-out sensitivity analysis for gut microbiota on HSP.....4**

**Figure S4. MR leave-one-out sensitivity analysis for gut microbiota on ITP......5**

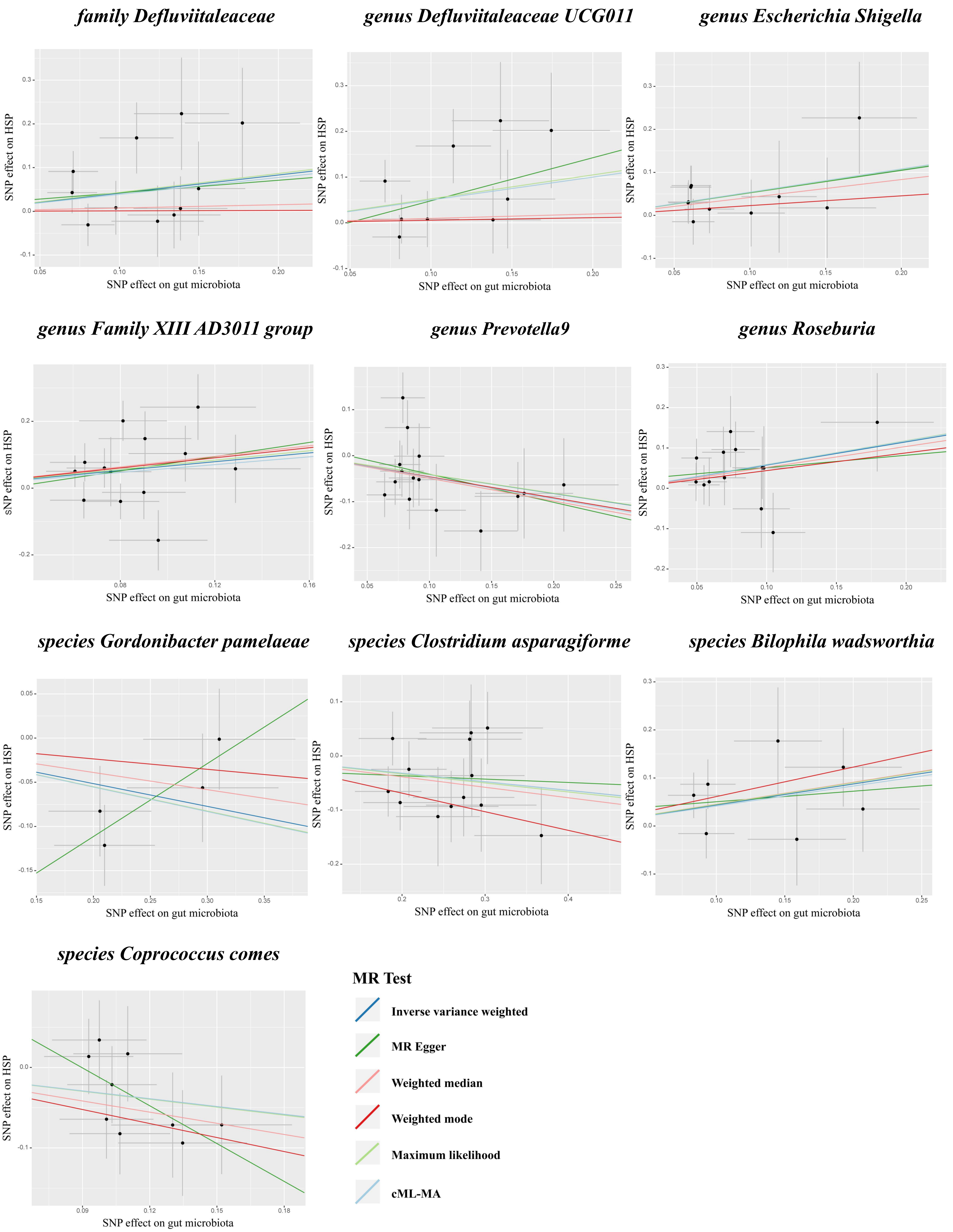


**Figure S1. Scatter plots for causal effect of gut microbiota on HSP.**

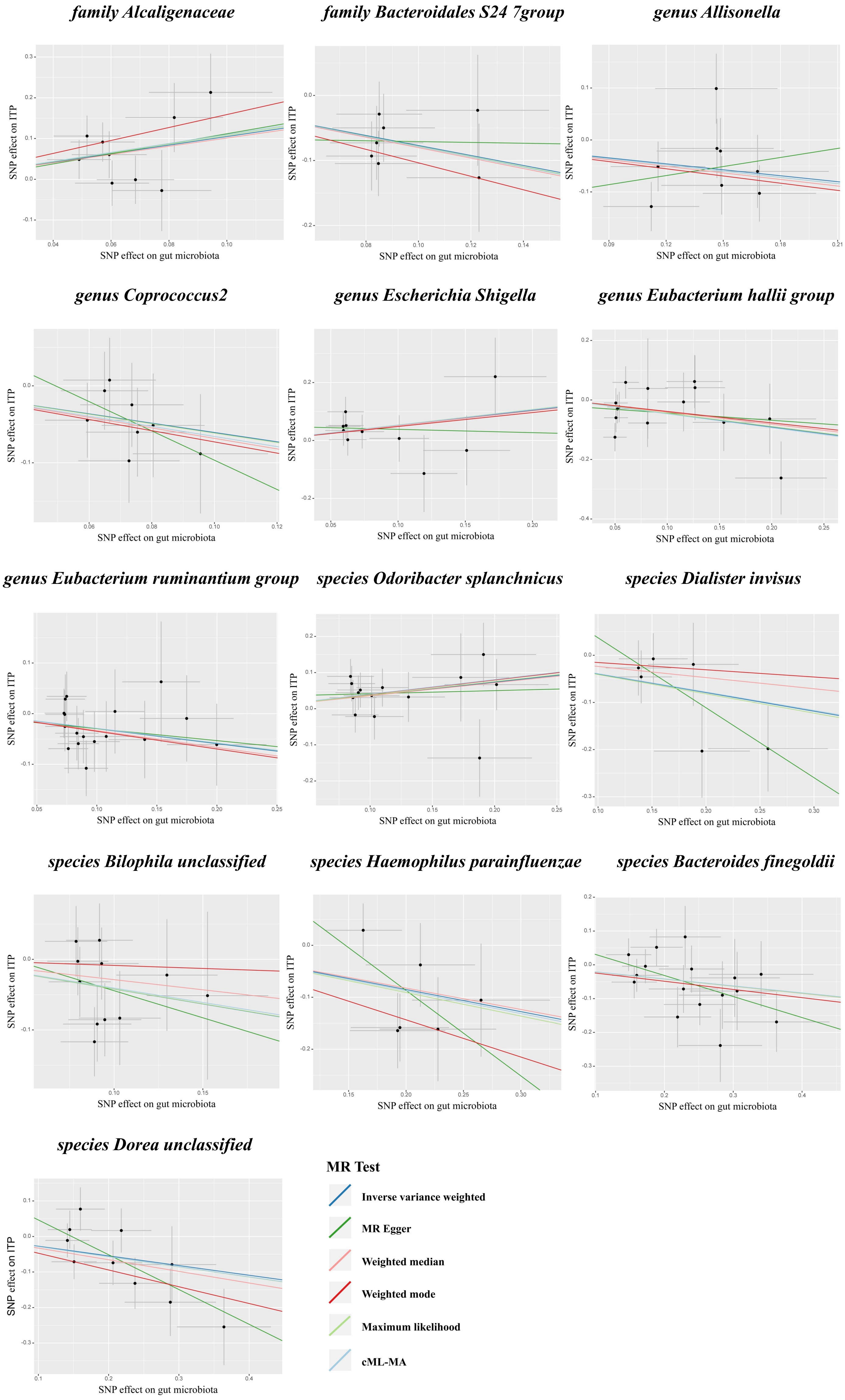


**Figure S2. Scatter plots for causal effect of gut microbiota on ITP.**

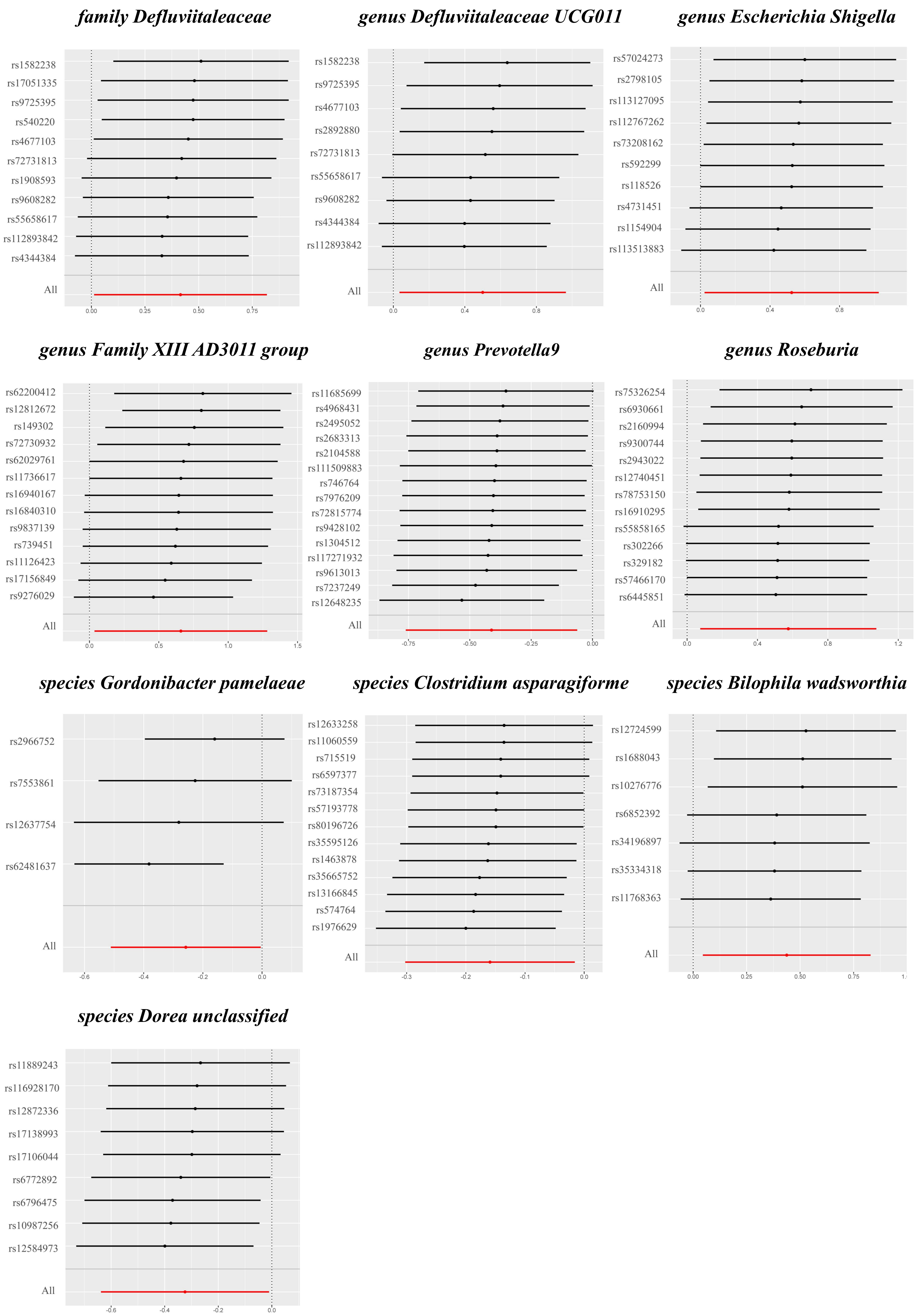


**Figure S3. MR leave-one-out sensitivity analysis for gut microbiota on HSP.**

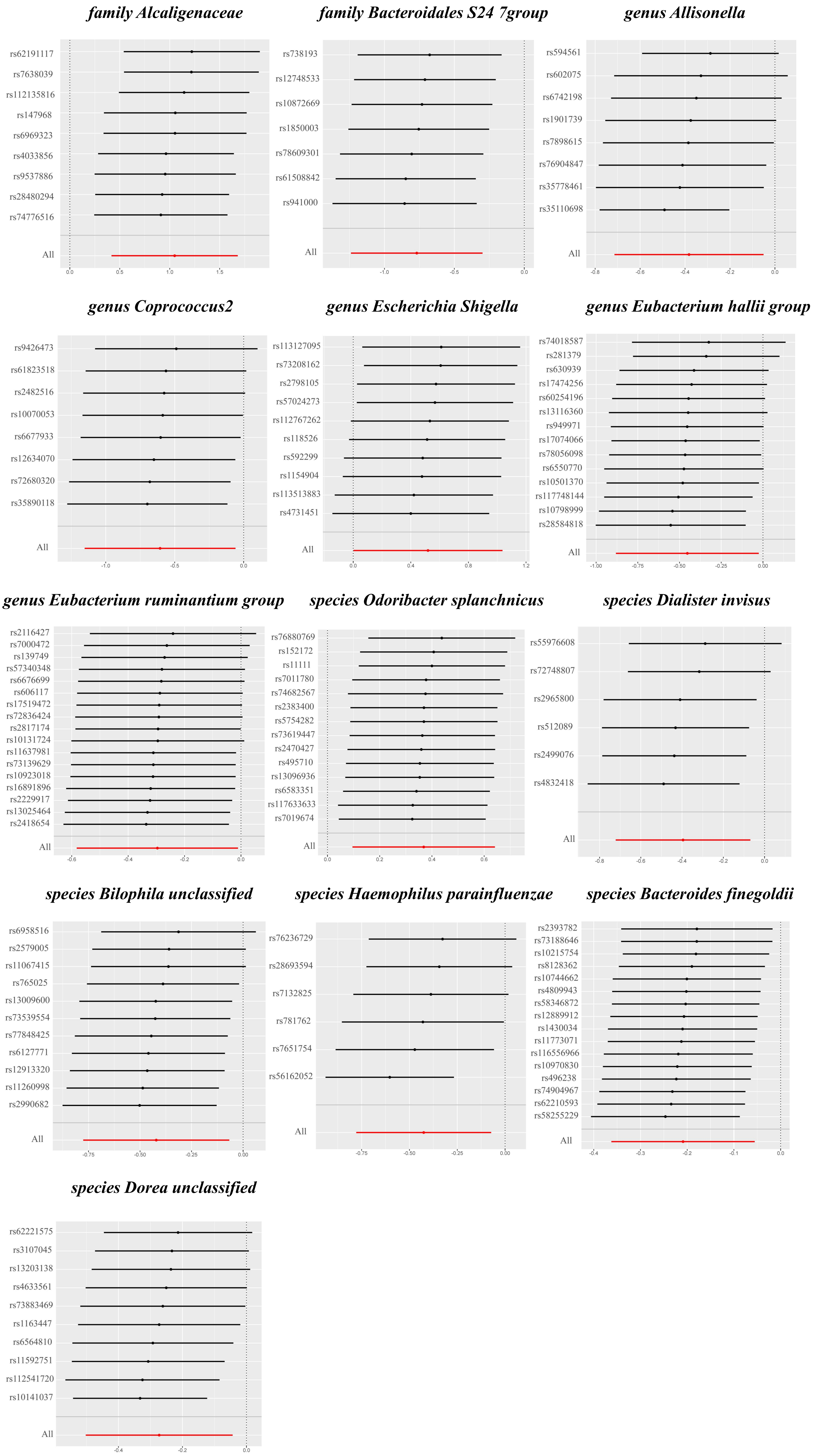


**Figure S4. MR leave-one-out sensitivity analysis for gut microbiota on ITP.**
